# Reporting of adverse events of treatment interventions in multiple myeloma: an overview of systematic reviews

**DOI:** 10.1007/s00277-023-05517-7

**Published:** 2023-11-08

**Authors:** Maria Mainou, Konstantinos I. Bougioukas, Konstantinos Malandris, Aris Liakos, Philippos Klonizakis, Ioannis Avgerinos, Anna-Betinna Haidich, Apostolos Tsapas

**Affiliations:** 1 Clinical Research and Evidence-Based Medicine Unit, Second Medical Department, Aristotle University of Thessaloniki, Thessaloniki, Greece; 2 Department of Hygiene, Social-Preventive Medicine and Medical Statistics, School of Medicine, Faculty of Health Sciences, Aristotle University of Thessaloniki, University Campus, 54124 Thessaloniki, Greece; 3 Adult Thalassemia Unit, Second Department of Internal Medicine, Hippokration Hospital, Thessaloniki, Greece; 4 Diabetes Centre, Second Medical Department, Aristotle University of Thessaloniki, Thessaloniki, Greece; 5 Harris Manchester College, University of Oxford, Oxford, UK

**Keywords:** Multiple myeloma, Overview, Systematic review, Adverse events

## Abstract

The present study is an overview of systematic reviews focusing on adverse events of antimyeloma treatments. It provides a systematic description of adverse events as they are reported in the systematic reviews as well as a critical appraisal of included reviews. We conducted a comprehensive literature search in the most widely used electronic databases looking for systematic reviews that had an adverse event of an antimyeloma treatment intervention as primary outcome. Two independent reviewers conducted selection of included studies and data extraction on predesigned online forms and assessed study quality using AMSTAR 2. Overall corrected covered area (CCA) was calculated to examine the overlap of primary studies across systematic reviews. After screening eligible studies, 23 systematic reviews were included in this overview. Seven reviews with overall CCA of 14.7% examined cardiovascular adverse events of different drugs, including immunomodulatory drugs and proteasome inhibitors (mainly carfilzomib). Nine focused on infections, presenting with overall CCA of 5.8%, each one focused on a different drug or drug class. Three studied thromboembolism in patients treated either with lenalidomide, any immunomodulatory drug, or with daratumumab and had an overall CCA equal to 1.5%. Four more reviews focused on bortezomib-associated neurotoxicity, carfilzomib-associated renal toxicity, or second primary malignancies as an adverse event of lenalidomide or anti-CD38 monoclonal antibody treatment. The quality of included studies as judged by AMSTAR 2 was mostly critically low. Absence of a priori registered protocol and formal assessment of risk of bias of included primary studies were the most common shortcomings. Reporting of antimyeloma drug-associated toxicity is supported by multiple systematic reviews; nevertheless, methodological quality of existing reviews is mostly low.

**Supplementary Information:**

The online version contains supplementary material available at 10.1007/s00277-023-05517-7.

## Background

Multiple myeloma is a hematologic malignancy with emerging treatment options that have improved the survival of patients through the last years, however remains uncurable and demands extended periods of treatment. Several drugs have been studied and are currently under investigation in a variety of clinical trials, most of them in combination of doublets or triplets, in different stages of the disease and with various results [1, 2]. The availability of a lot of treatment options that, in most cases, have not been appropriately compared head-to-head with each other, creates a state of uncertainty in clinical decision-making, especially in the setting of relapsed/refractory myeloma [3, 4].

Most of the drug regimens used in myeloma have a characteristic profile of adverse events that can inform and alter clinical decision-making process. Several published systematic reviews have studied adverse events of multiple myeloma drugs and this study aims to systematically report and appraise the quality of these reviews [5].

Research of adverse events can be a complicated process, as primary reports of clinical trials as well as systematic reviews and meta-analyses usually focus on efficacy of treatment interventions, underreporting harms [6, 7].

The present study provides a review of the adverse events presenting because of multiple myeloma treatment, usually affecting the quality of patients’ life. Based on the methodology of overviews of reviews [8], it reports all relevant systematic reviews focusing on adverse events, highlighting any lack of evidence. Additionally, the systematic approach and reporting of the therapeutic interventions in multiple myeloma with emphasis on harms may reveal any evidence of advantage or disadvantage of certain drugs based on their adverse events profile, informing clinical practice.

## Objectives

The objective of this study is to present all systematic reviews focusing on adverse events due to antimyeloma treatments, the way of looking for and presenting of adverse events, and the use of appropriate methods to synthesize the results, as well as reproducibility of results.

## Methods

A protocol of the current study is available and was designed a priori, but not officially registered. We report our results using the PRIO-harms tool, PRIO for abstract, and the PRIOR statement [9–11] for overviews of reviews.

### Criteria for considering reviews for inclusion

We included all systematic reviews focusing on adverse events of multiple myeloma treatment interventions. We included only systematic reviews, identified as such in title or abstract, that searched at least two different electronic databases and had as primary outcome an adverse event of an antimyeloma treatment intervention. We excluded all systematic reviews with both efficacy and toxicity outcomes when identified as such in the title as we wanted to focus only on harms. We also excluded all reports not written in English.

As already known, adverse events differ according to antimyeloma medication and can include infections, cardiotoxicity, peripheral neuropathy, and thromboembolism. These were the main adverse events expected. We did not specify outcomes of interest beforehand, as we wanted to include all potential adverse events.

As adverse events are not only reported in randomized controlled trials (RCTs) but in observational studies as well, in which longer follow-up can be more informative for rare events, we included systematic reviews of RCTs and observational studies with no restriction. The inclusion of observational studies is welcomed in the study of adverse events, while randomized controlled trials are the ideal study design when studying efficacy of a therapeutic intervention.

### Search methods for identification of reviews

We conducted a comprehensive search strategy in the most used electronic databases (Medline, Cochrane Library, Epistemonikos.org) as well as grey bibliography by handsearching references of included reviews. Day of the last search was November 4, 2022, in all databases. After completion of the study, we conducted a supplementary search on September 10, 2023, to include any recently published articles.

A full electronic search strategy in the three databases is available in the Appendix. We used appropriate filters for systematic reviews but not for adverse events as they are not inclusive enough and are not usually recommended.

### Data collection and analysis

#### Selection of reviews

All identified studies were imported into an online platform (DistillerSR®) that supports study selection and data extraction by two independent reviewers at the same time. Two independent reviewers screened potentially relevant reviews first by title and abstract and then in full text. Any disagreements between the reviewers were resolved through discussion.

#### Data extraction and mapping of primary study overlap

We extracted data from included systematic reviews in predesigned online forms that enabled two independent reviewers working at the same time. We collected data that included study’s research question, drug or drug combinations used, outcomes, characteristics of included primary studies, and population characteristics (patients with newly diagnosed myeloma or relapsed/refractory etc.). We also extracted relevant information about methodology used in systematic reviews: detailed search strategy, electronic databases searched, process of study selection, data extraction and assessing risk of bias in included studies, and use of statistics if meta-analysis was present. Any disagreements between the reviewers were resolved by consensus.

We created citation matrices to identify and visualize the degree of primary study overlap according to relevant methodology [12, 13]. We assessed systematic reviews with the same scope for overlapping by calculating overall CCA (corrected covered area) pairwise and by outcome (same adverse event) taking into account the chronological structural missingness (i.e., missing data of the matrix because primary studies were published after the conduct of a specific SR; therefore, it was not possible to be included in the review) [14].

#### Assessment of methodological quality of included reviews

We critically appraised the quality of the systematic reviews using AMSTAR 2 [15], in duplicate, using custom-designed online forms in DistillerSR®. Critical domains according to AMSTAR 2 original publication include protocol registration, adequacy of literature search, list of excluded studies with reasons, risk of bias of included studies, appropriateness of meta-analysis performed, and publication bias. We also reported if appropriate methods to evaluate the quality of individual studies were used in the systematic reviews (for example the Cochrane Risk of Bias tool for RCTs and the Newcastle Ottawa Scale or ROBINS-1 for non-randomized studies). These tools have a fixed set of domains of bias to be evaluated and judgement on quality is decided accordingly [16–18]. Finally, we recorded if GRADE approach for evaluating overall quality of evidence was available in the included studies. GRADE approach uses five domains to rate the quality of evidence from high to very low; these are as follows: risk of bias, inconsistency, indirectness, imprecision, and publication bias. Results of this assessment are usually presented in a summary of findings table, separately for every outcome [19].

#### Data synthesis

We present the results of this overview in a table with all included studies. We report summary measures of outcomes of interest as extracted from relevant reviews. Conducting a meta-analysis was beyond the scope of this overview, mainly due to high heterogeneity. Different drugs had different adverse events, and these could not be grouped together. We present harms reported in each study using outcome measures originally calculated in primary studies (mainly event rate (ER)/incidence, odds ratios (OR)/risk ratios (RR) for binary outcomes).

## Results

**Fig. 1:**
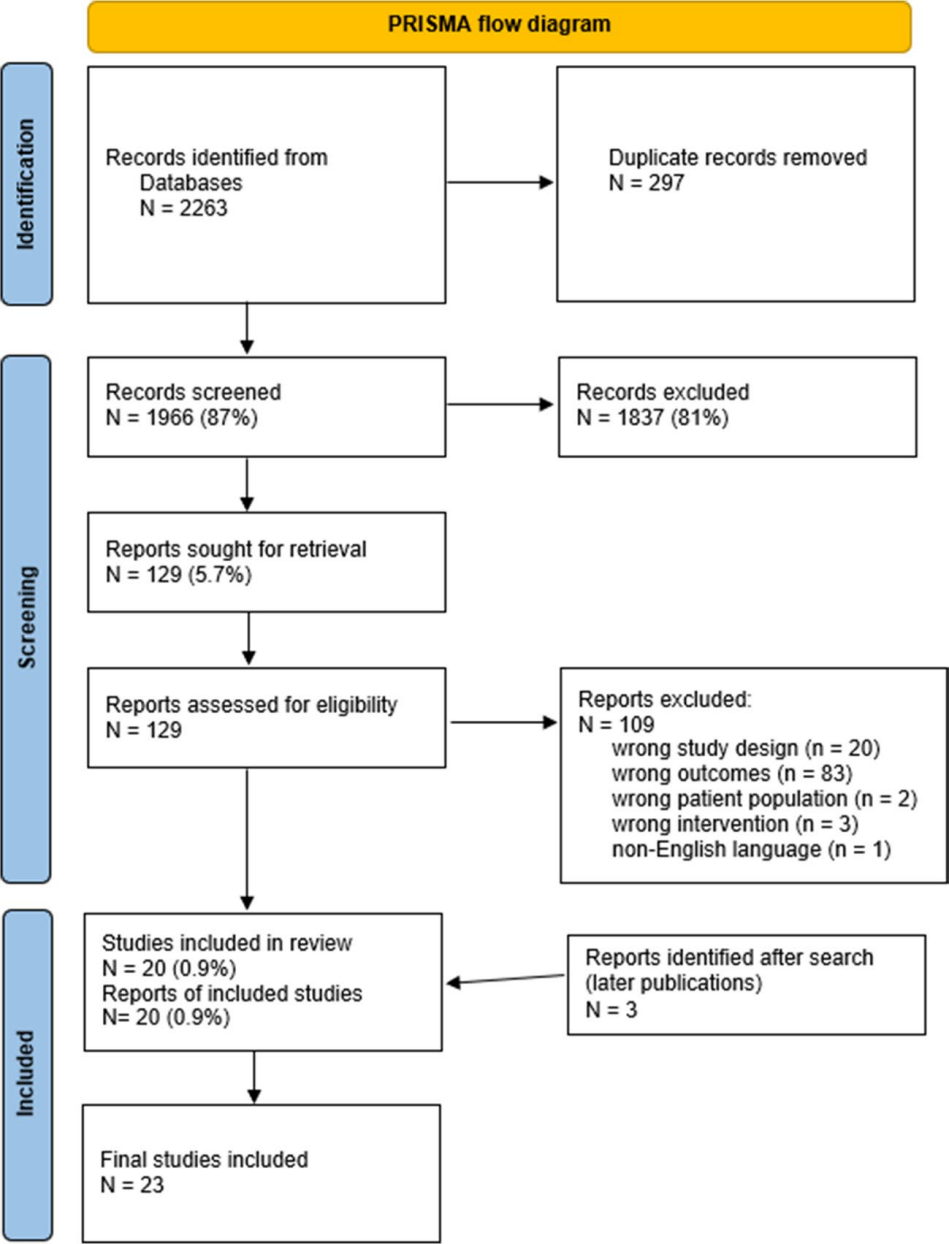
PRISMA flow diagram

Our initial search ended up with 2263 references. After deduplication, we screened by title and abstract 1966 records and 1837 were excluded for several reasons. Therefore, we included 129 reports for full-text screening. One hundred and nine reports were excluded (20 due to wrong study design, 83 due to efficacy outcomes, three for intervention of no interest (no antimyeloma drugs), and two for wrong patient population and one article was not in English). A full list of excluded studies after reading full text, with reasons for exclusion, is provided in the Appendix. After completion of the study, we run an updated search for recently published articles and three more systematic reviews were identified and included in this overview. Finally, 23 systematic reviews were included. The study selection process is depicted in flow diagram (Fig. 1) according to PRISMA [20].

### Description of included reviews

Twenty-three systematic reviews, including from 4 to 92 primary studies, are reported in this overview. Characteristics of included systematic reviews are described in Table 1. Thirteen of them included only randomized controlled trials (RCTs). Nine (45%) included RCTs and observational studies (prospective cohort studies) and one study included case-control studies as well. All but two systematic reviews included a meta-analysis as well, while one study included a network meta-analysis.

**Table 1:** Characteristics of included systematic reviews

Author, year	Population	Intervention of interest	Adverse event of focus	No of studies	No of patients	Type of studies included	GRADE reported	Use of Specific guidance for harms reported	Meta-analysis performed
1. Ball, 2020	NDMM/RRMM	Carfilzomib	Kidney toxicity	4	2954	RCT	No	No	Yes
2. Balmaceda, 2021	NDMM/RRMM	All anti-myeloma drugs	Infections	31	8680	RCT	No	No	Yes
3. Carrier, 2011	NDMM/RRMM	IMIDs	Thromboembolism	71	5690	RCT and cohort	No	No	Yes
4. Chakraborty, 2020	NDMM/RRMM	Lenalidomide	Thromboembolism	51	9069	RCT and cohort	No	No	Yes
5. Chen, 2018	NDMM/RRMM	IMIDs	Infections	92	19876	RCT, Cohort, case-control	Yes	No	Yes
6. Das, 2021	NDMM/RRMM	IMIDs/Pis	Cardiovascular/cardiotoxicity	20	10373	RCT	Yes	No	Yes
7. Latif, 2021	NDMM/RRMM	Carfilzomib	Cardiovascular/cardiotoxicity	42	5583	RCT and cohort	Yes	No	Yes
8. Li, 2019	NDMM/RRMM	Bortezomib	Neuropathy	23	8218	RCT	No	No	No
9. Ling, 2020	NDMM/RRMM	Ixazomib	Cardiovascular/cardiotoxicity	20	1715	RCT and cohort	No	No	Yes
10. Mian, 2023	NDMM/RRMM	Anti-CD38 MA	Second primary malignancies	10	4980	RCT	Yes	No	Yes
11. Palumbo, 2014	NDMM	Lenalidomide	Second primary malignancies	7	3254	RCT	No	No	Yes
12. Rahman, 2021	NDMM/RRMM	Carfilzomib	Cardiovascular/cardiotoxicity	4	2954	RCT	No	No	Yes
13. Reynolds, 2023	RRMM	Bispecific antibodies	Infections	20	1807	Clinical trials and cohort	No	No	Yes
14. Shah, 2018	NDMM/RRMM	Carfilzomib	Cardiovascular/cardiotoxicity	25	4164	RCT and cohort	No	No	Yes
15. Teh, 2016	NDMM/RRMM	IMIDs/Pis	Infections	30	13105	RCT	Yes	No	Yes
16. Vassilopoulos, 2022	NDMM/RRMM	Anti-CD38 MA	Infections	11	5316	RCT	No	No	Yes
17. Wang, 2020	NDMM/RRMM	Daratumumab	Thromboembolism	6	3802	RCT	No	No	Yes
18. Waxman, 2018	NDMM/RRMM	Carfilzomib	Cardiovascular/cardiotoxicity	24	2594	RCT and cohort	No	No	Yes
19. Wongsaengsak, 2020	NDMM/RRMM	Carfilzomib	Infections	4	2954	RCT	No	No	Yes
20. Wu, 2021	RRMM	Selinexor	Infections	7	578	RCT and cohort	No	No	No
21. Yarlagadda, 2021	NDMM/RRMM	Daratumumab	Infections	9	4752	RCT	No	No	Yes
22. Ying, 2017	NDMM/RRMM	Lenalidomide	Infections	11	3210	RCT	No	No	Yes
23. Zhao, 2018	RRMM	Carfilzomib	Cardiovascular/cardiotoxicity	8	2607	RCT and cohort	No	No	Yes

*Anti-CD38 MA* anti-CD38 monoclonal antibodies, *IMIDs* immunomodulatory drugs, *NDMM* newly diagnosed multiple myeloma, *PIs* proteasome inhibitors, *RCT* randomized controlled trials, *RRMM* relapsed/refractory multiple myeloma

**Fig. 2:**
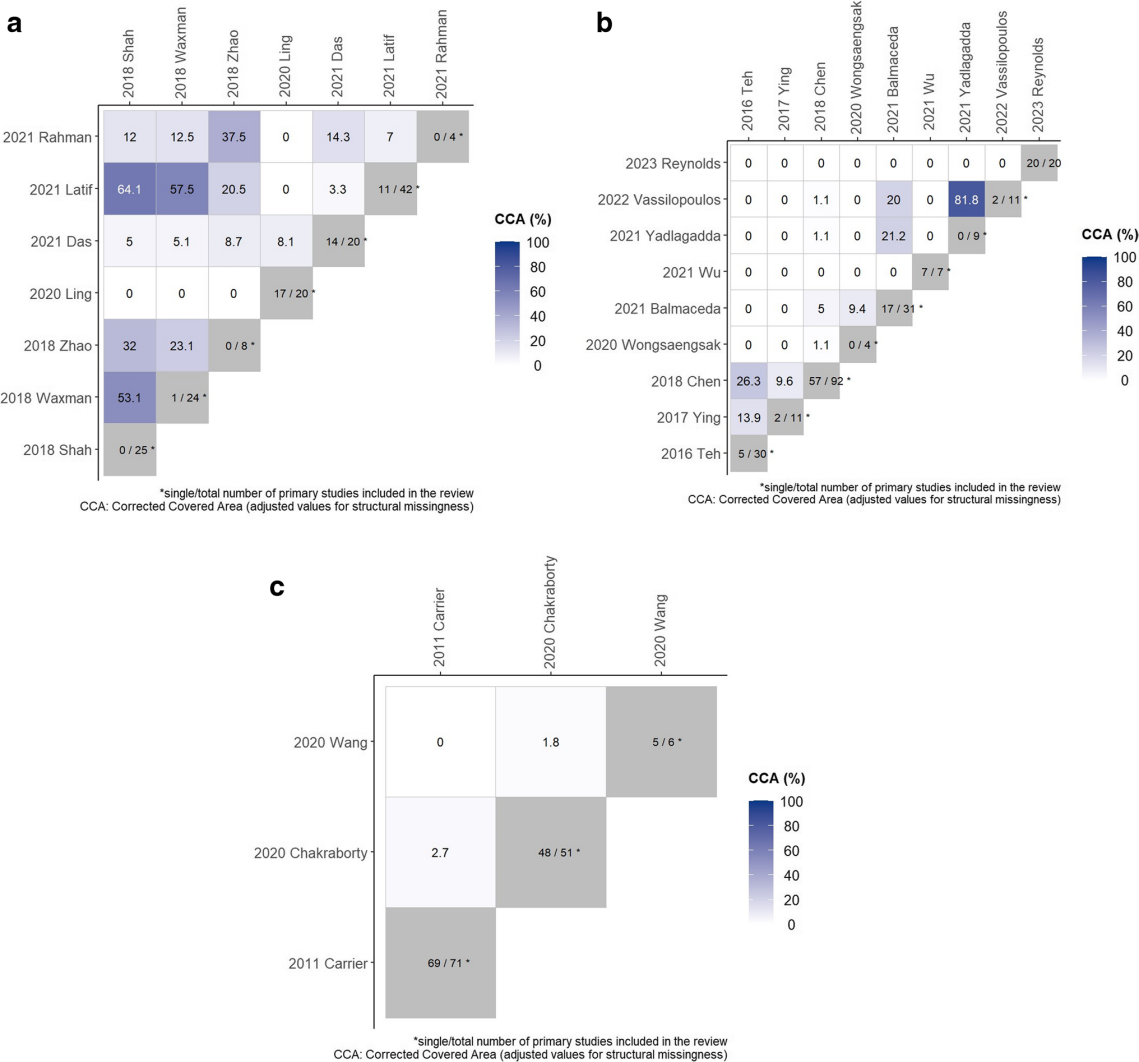
**a** Heatmap for visualization of overlap of included studies in reviews examining cardiotoxicity outcomes. The degree of overlap of primary studies between pairs of reviews is indicated by the value of the corrected covered area (CCA) index (CCA = 0% represents no overlap of primary studies [white color], CCA = 100% represents complete overlap of primary studies between the SRs [deep blue color]). Additionally, the plot presents the single/total number of primary studies included in each review in the diagonal tiles. A single primary study is exclusively included in only one SR (appeared in a single review) in the overview. **b** Heatmap for visualization of overlap of included studies in reviews examining infection outcomes. The degree of overlap of primary studies between pairs of reviews is indicated by the value of the corrected covered area (CCA) index (CCA = 0% represents no overlap of primary studies [white color], CCA = 100% represents complete overlap of primary studies between the SRs [deep blue color]). Additionally, the plot presents the single/total number of primary studies included in each review in the diagonal tiles. A single primary study is exclusively included in only one SR (appeared in a single review) in the overview. **c** Heatmap for visualization of overlap of included studies in reviews examining thromboembolism outcomes. The degree of overlap of primary studies between pairs of reviews is indicated by the value of the corrected covered area (CCA) index (CCA = 0% represents no overlap of primary studies [white color], CCA = 100% represents complete overlap of primary studies between the SRs [deep blue color]). Additionally, the plot presents the single/total number of primary studies included in each review in the diagonal tiles. A single primary study is exclusively included in only one SR (appeared in a single review) in the overview

Outcomes measures of included studies are presented in summary in Table 2. Seven (35%) reviews examined cardiovascular adverse events/cardiotoxicity. Five of them specifically focused on carfilzomib cardiotoxicity, one on ixazomib, and one on all proteasome inhibitors (PIs) and immunomodulatory drugs. Overlap of primary studies across the systematic reviews focusing on cardiotoxicity was 14.7% based on the adjusted CCA (Fig. 2a), indicating some overlap among reviews. Specifically, Shah et al. (2018), Latif et al. (2021), and Waxman et al. (2018), the three reviews that present higher overlap all focused on the cardiotoxicity of carfilzomib and included RCTs and observational studies in their review. Zhao et al. (2018) had a similar question but focused on the setting of relapsed myeloma and Rahman et al. (2021) only included RCTs. Ling et al., having almost no overlap with other reviews, had a different intervention under investigation, which was cardiotoxicity of ixazomib. Finally, Das et al. had only a small degree of overlapping as it included all RCTs of proteasome inhibitors and immunomodulatory drugs.

**Table 2:** Outcome measures

Author, year	Intervention of interest	Adverse event studied	Outcomes measure	Results
1. Ball, 2020	Carfilzomib	Kidney toxicity	RR for kidney toxicity/grade 3–5 toxicity	1.79 (1.43–2.23) *I*^2^ = 39% Grade 3–5: 2.29 (1.59–3.3) *I*^2^ = 24%
2. Balmaceda, 2021	All antimyeloma drugs	Infection	Incidence of high-grade infection	NDMM 1.7% (1.3–2) *I*^2^ = 98%
				RRMM 1.5% (1–2) *I*^2^ = 99.53%
				Maintenance 0.4% (0.2–0.6) *I*^2^ = 98.59%
3. Carrier, 2011	IMIDs (lenalidomide, thalidomide)	Thromboembolism	Incidence per 100 patient-cycles/months	Thalidomide + Dex, NDMM = 4.1 (2.8–5.9)/RRMM = 0.8 (0.1–2.1)
				Lenalidomide + Dex, NDMM = 0.8 (0.07–2)/RRMM = 0.7 (0.4–0.9)
				Different rates with prophylaxis
4. Chakraborty, 2020	Lenalidomide	Thromboembolism	Incidence of VTE	6% (5.1–7.1) *I*^2^ = 66.4%
5. Chen, 2018	IMIDs (lenalidomide, thalidomide, pomalidomide)	Infection	Incidence for serious infection in different settings	Range 7–23%, from observational studies 8.8% (6.9–10.7) *I*^2^= 74.5%
			RR of serious infection	ASCT-ineligible, 1.59 (1.31–1.93) *I*^2^ = 0% ASCT-eligible, 0.82 (0.72–0.94) *I*^2^ = 45.6% Maintenance, 1.59 (1.26–2.01) *I*^2^ = 0% RRMM, 1.38 (1.08–1.78) *I*^2^ = 0%
6. Das, 2021	IMIDs/PIs	Cardiotoxicity	OR of high-grade cardiotoxic events	With IMIDs, 2.05 (1.3–3.26) *I*^2^ = 10%
				With PIs, 1.67 (1.17–2.40) *I*^2^ = 0%
				Network, carfilzomib 2.68 (1.63–4.4)/bortezomib 1.18 (0.73–1.92)/ixazomib 1.56 (0.84–2.9) for all-grade cardiotoxicity
7. Latif, 2021	Carfilzomib	Cardiotoxicity, Hypertension	Incidence of all grade cardiotoxicity/high-grade, all grade/high-grade hypertension, heart failure, edema, ischemia	All grade cardiotoxicity 8.9% (6.6–11.8) *I*^2^ = 83%/high-grade 4.4% (3.6–5.4) *I*^2^ = 17%
				All grade hypertension 13.2% (9.8–17.6) *I*^2^ = 88%/high grade 5.3% (3.1–8.9) *I*^2^ = 90%
				Heart failure 5.1% (2–12) *I*^2^ = 93%
				Edema 20.7% (12.4–32.5) *I*^2^ = 89%
				Ischemia 4.6% (1.5–13.5) *I*^2^ = 67%
8. Li, 2019	Bortezomib	Neuropathy	Incidence of all grade and severe PN	Range of all grade, 8.4–80.5% (median 37.8%) Severe, 1–33.2% (median 8%)
9. Ling, 2020	Ixazomib	Cardiovascular	Event rate of CVAE/high-grade CVAE	11.2% (7.1–15.2) *I*^2^ = 90.81%/high grade 3.7% (2.1–5.2) *I*^2^ = 69.43%
			RR	1.098 (0.873–1.380)/high grade 1.679 (1.078–2.615) *I*^2^ = 0%
10. Mian, 2023	Anti-CD38 monoclonal antibodies	Second primary malignancies	Peto OR of developing second primary malignancies	All, 1.53 (1.20–1.95) *I*^2^ = 0%
				Non-melanoma cutaneous cancers, 1.77 (1.25–2.51) *I*^2^ = 0%
				Solid, 1.28 (0.85–1.95) *I*^2^ = 14%
				Hematologic, 1.86 (0.81–4.27) *I*^2^ = 0%
11. Palumbo, 2014	Lenalidomide	Second primary malignancies	Incidence at 5 years	6.9% (5.3–8.5)
			HR	1.55 (1.03–2.34)
12. Rahman, 2021	Carfilzomib	Heart failure	RR for all grade / grade 3-5 HF	2.34 (1.66–3.32) *I*^2^ = 9% Grade 3–5, 2.69 (1.77–4.09) *I*^2^ = 0%
13. Reynolds, 2023	Bispecific antibodies	Infections	ER for all-grade and grade ≥ 3 infections	All-grade, 56% (0.48–0.65) *I*^2^ = 92% Grade ≥ 3, 21% (0.15–0.27) *I*^2^ = 89%
				Bispecific monotherapy all-grade, 51% (0.38–0.63) *I*^2^ = 93% Bispecific combination therapy all-grade, 52% (0.43–0.61) *I*^2^ = 91%
				BCMA-targeting bispecific all-grade, 71% (0.49–0.92) *I*^2^ = 91% Non-BCMA-targeting bispecific all-grade, 55% (0.42–0.68) *I*^2^ = 88%
			Infection-related mortality	3% (0.01–0.04)
14. Shah, 2018	Carfilzomib	Cardiotoxicity	Event rate for all grade/ high-grade cardiotoxicity	All grade, 8.68% (6.13–11.59) *I*^2^ = 79.95% High-grade, 4.92% (3.91–6.02) *I*^2^ = 22.37%
			OR	All grade, 2.03 (1.19–3.46) *I*^2^ = 44.93% High grade, 2.04 (1.31–3.17) *I*^2^ = 0%
15. Teh, 2016	IMIDs/PIs	Infection	Event rate for severe infection	IMIDs non-transplant, 13.4%/transplant 22.4%/maintenance 10.5%/RRMM 16.6%
			RR of severe infection	IMIDs non-transplant, 1.74 (1.43–2.12) *I*^2^ = 0%/transplant, 0.76 (0.67–0.86) *I*^2^ = 77%/maintenance, 1.74 (1.4–2.26) *I*^2^ = 1%/RRMM 1.51 (1.18–1.93) *I*^*2*^ = 22% PIs transplant, 1.12 (0.89-1.4) *I*^2^ = 70%
16. Vassilopoulos, 2022	Anti-CD38 monoclonal antibodies	Infections	RR for any grade and severe infections	Any grade and severe, 1.27 (1.17–1.37and 1.14–1.41) *I*^2^ = 59.03% and O%, respectively
				Pneumonia any, 1.39 (1.12–1.72) *I*^2^ = 49.80% Pneumonia severe, 1.38 (1.09–1.75) *I*^2^ = 37.66%
				URI any, 1.51 (1.35–1.70) *I*^2^ = 0%
				VZV reactivation, 3.86 (0.66–22.50) *I*^2^ = 0%
				Death, 1.35 (0.76–2.40) *I*^2^ = 0%
			Incidence	Any grade, 77% (95% CI, 68–86%) *I*^2^ = 95.09% Severe, 28% (95% CI, 23–34%) *I*^2^ = 89.03%
17. Wang, 2020	Daratumumab	Thromboembolism, Thrombocytopenia, GI bleeding	RR	VTE, 0.6 (0.4–0.91) *I*^2^ = 0%
				ATE, 0.8 (0.48–1.33) *I*^2^ = 0%
				Thrombocytopenia 3/4, 1.14 (0.94–1.38) *I*^2^ = 39.3%
				GI bleeding, 1.32 (0.38–4.65) *I*^2^ = 0%
18. Waxman, 2018	Carfilzomib	Cardiovascular	Event rate for all grade and high-grade CVAE	All grade, 18.1% (13.5–23.3) *I*^2^ = 87.4% High grade, 8.2% (5.9–10.7) *I*^2^ = 71.6%
			RR for all grade and high-grade CVAE	All grade, 1.8 (1.4–2.2) *I*^2^ = 14.8% High grade, 2.2 (1.6–2.9) *I*^2^ = 0%
19. Wongsaengsak, 2020	Carfilzomib	Infection	RR of serious infection	1.40 (1.17–1.69) *I*^2^ = 57% Respiratory tract infection, 1.3 (1.12–1.5) *I*^2^ = 0%
20. Wu, 2021	Selinexor	Infection	Incidence of severe infection	17.3%
21. Yarlagadda, 2021	Daratumumab	Infection, Neutropenia, Lymphopenia	RR	Pneumonia, 1.58 (1.36–1.83) *I*^2^ = 57%
				URTI, 1.5 (1.33–1.69) *I*^2^ = 63%
				Neutropenia 1.27 (1.19–1.36) *I*^2^ = 84%
				Lymphopenia 1.45 (1.21–1.72) *I*^2^ = 44%
22. Ying, 2017	Lenalidomide	Infection	Incidence of high-grade infection	14.32% (12.08–16.9) *I*^2^ = 52.3%
			OR	OR, 2.23 (1.71–2.91) *I*^2^ =0%
23. Zhao, 2018	Carfilzomib	Cardiotoxicity	Event rate for all grade congestive heart failure/ischemic heart disease	CHF, 5.5% (4.3–6.9) *I*^2^ = 16%; IHD, 2.7% (1.1–6.7)
			Peto-OR	CHF, 2.33 (1.56–3.48) *I*^2^ = 29%; IHD, 1.31 (0.79–2.18) *I*^2^ = 30%

*ASCT* autologous stem cell transplantation, *ATE* arterial thromboembolism, *CHF* congestive heart failure, *CVAE* cardiovascular adverse events, *Dex* dexamethasone, *ER* event rate, *GI* gastrointestinal, *HF* heart failure, *HR* hazard ratio, IHD ischemic heart disease, *IMIDs* immunomodulatory drugs, *NDMM* newly diagnosed multiple myeloma, *OR* odds ratio, *PIs* proteasome inhibitors, *PN* peripheral neuropathy, *RR* risk ratio, *RRMM* relapsed/refractory multiple myeloma, *URI* upper respiratory infection, *URTI* upper respiratory tract infection, *VTE* venous thromboembolism, *VZV* varicella-zoster virus

Nine reviews (39%) had infections as the main outcome of interest. Of them, one studied infections in patients treated with carfilzomib, one with daratumumab, one with selinexor, one with lenalidomide, one with immunomodulatory drugs (thalidomide/lenalidomide/pomalidomide), one with immunomodulatory drugs or proteasome inhibitors, one with anti-CD38 monoclonal antibodies, one with bispecific antibodies, and one included every anti-myeloma drug. The overall degree of overlap between reviews was low, based on adjusted CCA, which was 5.8% (Fig. 2b). Chen et al. and Teh et al. presenting with slightly higher overlap examined infections as an adverse of IMIDs (immunomodulatory drugs) (Chen) and IMIDs/PIs (Teh). However, one included only RCTs and one RCTs, cohort, and case-control studies. The highest overlap was between Vassilopoulos et al. and Yadlagadda et al., who both studied impact of anti-CD38 monoclonal antibodies on infection (Vassilopoulos both daratumumab and isatuximab and Yadlagadda only daratumumab) with difference in their date of search (January 2021 in Yadlagadda paper). The overall low degree of overlap in reviews focusing on infections can be explained mainly by the different interventions under investigation. Almost every SR studied a different anti-myeloma drug and some of them included RCTs only while others observational studies as well.

Three studies (15%) focused on thromboembolism as an adverse event of lenalidomide (one study), of all immunomodulatory drugs (one study) and of daratumumab (one study). In this case, overlap among the three reviews focusing on thromboembolism was low (adjusted CCA 1.5%, Fig. 2c), as the three of them had slightly different PICO (Population–Intervention–Comparator–Outcome) questions and one of them was published 9 years before the others. Neuropathy of bortezomib, kidney toxicity of carfilzomib, and second primary malignancies as an adverse event of lenalidomide or anti-CD38 monoclonal antibodies’ treatment were the main interests of the remaining included studies.

### Methodological quality of included reviews

Of the 23 studies, 20 (87%) reported results based on PRISMA guidelines for reporting of systematic reviews, while three studies did not use a formal guide. Only four systematic reviews used the GRADE approach for evaluating the overall quality of evidence and none of them mentioned the use of any guidance for reporting harms (PRISMA-harms, Prio-harms). Data and code for the meta-analysis performed were available by the reviewers only in four cases, where authors provided a data availability statement. One of them also mentioned that statistical codes used for meta-analysis are publicly available. Handling of missing data was reported in only two systematic reviews, that both mentioned contact of authors for data, and one of them excluding the study in question given that full data was after all not available. When meta-analysis was performed, random effects meta-analysis was used most of the time (in 14 out of 21 reviews). Three SRs used both fixed and random effects model in their meta-analysis, one used fixed or random effects depending on heterogeneity. Finally, one network meta-analysis used individual patient data.

AMSTAR 2 was performed for each study (Table 3). Most systematic reviews had a clear research question with inclusion criteria that included the components of PICO, although description of the comparator was not always applicable. However, only three studies had an a priori registered protocol. A comprehensive search strategy was mentioned in most reviews, duplicate study selection was performed in 19 out of 23 (83%) studies while data extraction in duplicate was conducted in 15 (65%). None of the studies reported a list of excluded studies but almost everyone adequately described included studies. Assessment of risk of bias was adequately performed (using appropriate tools such as the Cochrane Risk of Bias tool for RCTs and the Newcastle Ottawa Scale for non-randomized studies) in ten out of 22 studies (44%) that included RCTs and only in one out of nine studies that included cohort studies. Funding of primary studies was not assessed in any review but conflict of interest of review authors was usually reported appropriately. In case meta-analysis was performed, appropriate statistical methods were used for combination of results in more than half of the reviews but not always separately for RCTs and cohort studies. However, risk of bias was rarely accounted for by authors in the interpretation of the results. Heterogeneity was discussed and a possible explanation was explored if present in most studies and publication bias was investigated in almost every systematic review.

**Table 3:**
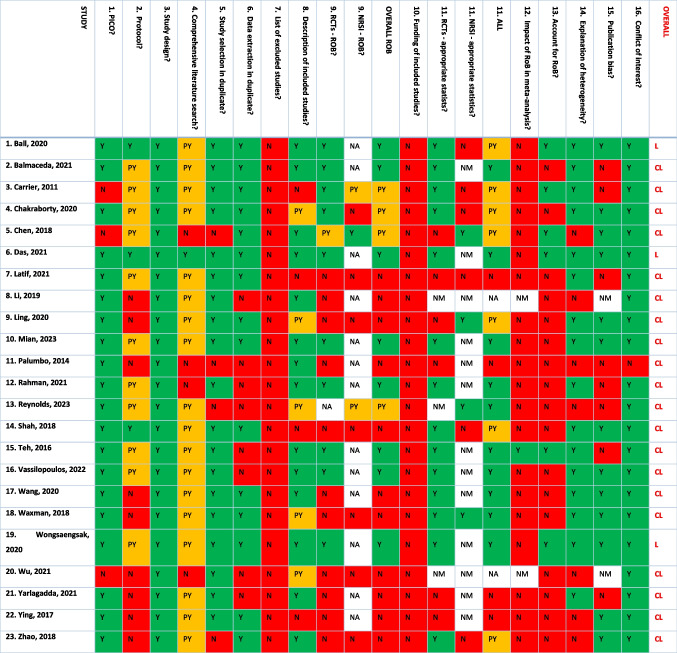
AMSTAR 2 assessment of included studies

Green indicates Y: Yes, orange indicates PY: Partial Yes, red indicates N: No. Last column represents overall quality of each systematic review

*CL* critically low, *L* low, *NA* not applicable (study does not include RCTs/NRSI), *NM* no meta-analysis conducted, *NRSI* non-randomized studies, *PICO* Population–Intervention–Comparator–Outcome, *RCTs* randomized controlled trials, *ROB* risk of bias

When rating the overall confidence in the results of each review, following examination of AMSTAR 2 critical domains, most studies (20 out of 23) were of critically low quality. Almost all our included reviews had more than one critical flaw, leading to critically low confidence in their results.

Grade methodology was used in four studies (17%), rating the overall quality of evidence in each study from moderate to low. We were not able to formally use GRADE in our overview as most of the included studies had not performed a proper risk of bias assessment.

### Effects of interventions

We identified seven (35%) reviews examining adverse events of carfilzomib. Five of them assessed cardiovascular adverse events. The most recent study by Rahman et al. [21] included all four available RCTs to date. Carfilzomib increased heart failure with a risk ratio of 2.34 (1.66–3.32), *I*^2^ = 9% compared to other treatments. Waxman [22], Shah [23], Zhao [24], and Latif [25] all focused on cardiotoxicity using data from RCTs and cohort studies. The event rate of congestive heart failure in Zhao et al. was 5.5% (4.3–6.9), *I*^2^ = 16%, and Peto-OR for congestive heart failure of any grade was 2.33 (1.56–3.48), *I*^2^ = 29%. Latif et al. reported an incidence of cardiotoxicity (including cardiac failure, ischemia, and arrest) of any grade 8.9% (6.6–11.8), *I*^2^ = 83%, and of heart failure 5.1% (2.0–12.0), *I*^2^ = 93%. In Shah et al., incidence of cardiotoxicity (including adverse cardiac events, such as acute coronary syndrome, myocardial infraction, and cardiac failure) of any grade was 8.7% (6.1–11.6), *I*^2^ = 79.9%, and OR 2.03 (1.19–3.46), *I*^2^ = 44.93%. In Waxman et al., RR was 1.8% (1.4–2.2), *I*^2^ = 14.8%. Kidney toxicity (mainly acute kidney injury, kidney impairment, toxic nephropathy, thrombotic microangiopathy or thrombotic thrombocytopenic purpura, increased creatinine, etc.) of carfilzomib reported in Ball et al. had a RR of 1.79 (1.43–2.23), *I*^2^ = 39%. Finally, total serious infections with carfilzomib had a RR of 1.40 (1.17–1.69), *I*^2^ = 57%, as reported by Wongsaengsak et al.

Lenalidomide alone was investigated in three systematic reviews. One included RCTs and cohort studies and two included only RCTs. Outcomes of interest were incidence of any infection in Ying et al. [26] of 14.32% (12.08–16.90), *I*^2^ = 52.3%, and a OR 2.23 (1.71–2.91), *I*^2^ = 0%, second primary malignancies with an event rate of 6.9% (5.3–8.5) and HR (hazard ratio) of 1.55 (1.03–2.34) in Palumbo et al. [27], and thromboembolism with an incidence of 6% (5.1–7.1), *I*^2^ = 66.4%, in Chakraborty et al. [28].

Immunomodulatory drugs were examined in two more systematic reviews. One included observational studies and RCTs of lenalidomide and thalidomide and focused on thromboembolism, reporting different event rates in patients with or without coagulation [29]. The latter included RCTs, cohort, and case-control studies of lenalidomide, thalidomide, and pomalidomide and focused on infections. IMIDs had an ER (event rate) of 7–23% depending on setting. In ASCT (Autologous Stem Cell Transplant) ineligible patients Chen et al. reported a RR of 1.59 (1.31–1.93), *I*^2^ = 0%; in ASCT eligible 0.82 (0.72–0.94), *I*^2^ = 45.6%; and in relapsed/refractory setting 1.38 (1.08–1.78), *I*^2^ = 0% [30].

Two reviews focused on studies with IMIDs and PIs. Das et al. reported cardiotoxicity with IMIDs with an OR 2.05 (1.3–3.26), *I*^2^ = 0%, and PIs 1.67 (1.17–2.4), *I*^2^ = 0% [31]. Teh et al. focused on incidence of infections and reported different RR according to setting. With IMiDs in non-transplant eligible patients, RR was 1.74 (1.43–2.12), *I*^2^ = 0%, while in transplant setting 0.76 (0.67–0.86), *I*^2^ = 77%. PIs had an infection RR of 1.12 (0.89–1.4), *I*^2^ = 70%, and in relapsed/refractory multiple myeloma (RRMM) IMIDs had a RR of 1.51 (1.18–1.93), *I*^2^ = 22% [32].

Daratumumab was studied in two systematic reviews, one focusing on thromboembolism (RR for venous thromboembolism (VTE) 0.6 (0.4–0.91), *I*^2^ = 0%, and arterial thromboembolism 0.8 (0.48–1.33), *I*^2^ = 0%, in Wang et al.) [33] and the second infection. In Yarlagadda et al., RR for pneumonia with daratumumab was 1.58 (1.36–1.83), *I*^2^ = 57%, and for upper respiratory tract infection 1.5 (1.33–1.69), *I*^2^ = 63% [34].

Anti-CD38 monoclonal antibodies (both daratumumab and isatuximab) were investigated by two more systematic reviews, one focusing on infections and the other on second primary malignancies. Vasilopoulos et al. [35] reported RR for any grade and severe infections with anti-CD38 antibodies of 1.27 (1.17–1.37and 1.14–1.41) *I*^2^ = 59.03% and O%, respectively. This RR for pneumonia was 1.39 (1.12–1.72), *I*^2^ = 49.80%, and for VZV reactivation 3.86 (0.66–22.50) *I*^2^ = 0%. Incidence of any grade of infection with the monoclonal antibodies was 77% (95% CI, 68%–86%) *I*^2^ = 95.09% [35]. The two monoclonal antibodies showed an OR of developing second primary malignancies of 1.53 (1.20–1.95) *I*^2^ = 0% in the systematic review of Mian et al. which was mainly due to non-melanoma cutaneous cancers (OR 1.77 (1.25–2.51) *I*^2^ = 0%) [36].

Wu et al. studied infection in patients treated with selinexor, including RCTs and cohort studies without meta-analysis, reporting an incidence of 17.3% [37]. Ling et al. explored cardiovascular adverse events associated with the use of ixazomib using RCTs and observational studies. They reported an event rate of 11.2% (7.1–15.2), *I*^2^ = 90.81%, and a RR 1.098 (0.873–1.380) in RCTs [38]. Bortezomib treatment had an event rate of neuropathy of any grade ranging from 8.4 to 80.5% in different studies as reported by Li et al., without a relevant meta-analysis [39]. Finally, incidence of infection in any patient with multiple myeloma treated with any anti-myeloma drug was examined by Balmaceda et al. and reported incidence and RR according to setting, with higher rates in newly diagnosed multiple myeloma [40].

Bispecific antibodies (BCMA targeting and non-BCMA targeting) were the newest drug category to be studied by Reynolds et al., with focus on infections. Their results coming from studies with no control group had an ER for all-grade infections 56% (0.48–0.65) *I*^2^ = 92% and for grade ≥ 3: 21% (0.15–0.27) *I*^2^ = 89% [41].

## Discussion

### Summary of main results

Adverse events of anti-myeloma drugs were the interest of this overview of systematic reviews. The main adverse events examined by the included systematic reviews were cardiotoxicity, infections, thromboembolism, kidney disease, peripheral neuropathy, and second primary malignancies. A multitude of drugs or drug classes constitute therapeutic options in multiple myeloma, with different profile of adverse effects.

Cardiotoxicity is a serious adverse event of carfilzomib examined in many studies. This has also been noted by a recent overview of systematic reviews [42]. This adverse event appears to be decisive in its use, with kidney toxicity and infection also noted. Regarding kidney toxicity, although not reported in detail in the systematic review by Ball et al. in their odds ratios, newer evidence has highlighted the incidence of thrombotic microangiopathy (TMA) in patients treated with carfilzomib [43, 44]. This is an adverse event of special interest that may lead to stopping treatment with this agent.

Infection is a well-reported adverse event, complicating treatment with almost every drug, mainly immunomodulatory drugs, proteasome inhibitors, anti-CD38 monoclonal antibodies, and selinexor but also bispecific antibodies and CAR-T cells. Infection rates differ by disease state (newly diagnosed/relapsed myeloma) and treatment regimens (triplet versus doublet combinations). Antibiotic prophylaxis with levofloxacin may limit the risk of infection, especially during the first cycles of treatment after diagnosis [45]. The role of antibiotic prophylaxis and more importantly appropriate vaccinations in multiple myeloma has been studied for many years but more detailed guidelines have been issued incorporating current knowledge [46–48]. Prophylactic agents, such as cotrimoxazole for pneumocystis infection and acyclovir or valacyclovir for varicella-zoster virus, play a pivotal role in preventing specific types of infections while on treatment with specific drugs (namely proteasome inhibitors and antibodies) [47–49]. While these agents are essential in clinical practice and have shown effectiveness in various studies, they were not described in detail in the reviews we included.

Thalidomide has historically been associated with a higher risk of thromboembolism compared to lenalidomide in certain contexts as mentioned in our included studies. However, with the global decline in thalidomide use and the increased application of prophylactic measures in patients treated with lenalidomide, the current landscape may present a different picture. This risk seems to depend on the disease phase as well as the exact agents used, for example, it may be different for lenalidomide used as monotherapy and different for doublet or triplet regimens. Recent guidelines and studies provide insights into patient stratification for prophylactic treatments while receiving these agents [50–52]. The best thromboprophylaxis regimen is not the same for every patient. However, as different tools have been developed to stratify the risk of thrombosis in multiple myeloma patients, considering individual risk factors like previous thromboembolism, comorbidities, central venous catheter, immobilization etc., as well as myeloma-related factors and chemotherapy regimen used, decision on thromboprophylaxis is becoming more informed [53–57]. Thus, although aspirin is recommended for low-risk patients, low molecular weight heparin has been widely used for those of higher risk, and lately direct oral anticoagulants (DOACs) are explored as more appealing treatment options [58].

Bortezomib-associated neuropathy is a well-recognized entity and several pathophysiological mechanisms have been proposed. While this adverse event has historically raised concerns, its clinical occurrence is notably reduced with current treatment approaches, such as subcutaneous administration and once-weekly dosing. Early dose-reduction or tapering of bortezomib further minimizes this risk. Since treatment of neuropathy after its occurrence is rather unsatisfactory, recognition of early symptoms is important as there are guidelines of dose reductions to prevent further damage [59–62]. Notably, thalidomide-associated peripheral neuropathy can be irreversible, whereas bortezomib-induced PNP is often of a milder grade (1–2) and is frequently reversible [63, 64]. However, thalidomide-associated neuropathy has not been reported in included reviews.

Prolonged treatment with lenalidomide, particularly in combination therapies, has been associated with an elevated risk of secondary primary malignancies. This risk factor becomes particularly salient in the context of maintenance treatments, which are increasingly being administered over extended durations or until the point of disease progression. In contrast, anti-CD38 antibodies have not been broadly implicated in the development of malignancies, apart from non-melanoma cutaneous cancers.

The degree of overlap among included reviews was generally low. This can be attributed to different research questions used by each study. We examined overlap separately for each outcome; however, heterogeneity was expected to be high, as different agents were investigated by each study. Additionally, different criteria for included studies in the reviews had been used. Many of them only included RCTs while others included observational studies as well, which is reasonable in the research of adverse events.

The quality of included systematic reviews as judged by AMSTAR 2 ranged from critically low to low. This was mainly due to lack of a registered protocol and lack of quality assessment of included studies. Although some systematic reviews including RCTs have used an appropriate tool for risk of bias assessment, only one study used the Newcastle Ottawa Scale for evaluating the quality of cohort studies. The GRADE approach was not widely used in the included systematic reviews. Also, none of the reviews included a list of excluded studies with reasons.

Furthermore, while most systematic reviews reported results according to PRISMA guideline, none of them reported adverse events according to PRISMA-harms [6] despite the fact that seventeen out of twenty included SRs were published after the publication of PRISMA-harms. Also, reproducibility of systematic reviews and meta-analyses was problematic as data availability was mentioned only in two systematic reviews [65].

### Potential biases in the overview process

We must acknowledge several limitations in our study, with the primary one being the lack of a formally registered protocol. Our analysis did not include systematic reviews not written in English, and we focused exclusively on reviews that specifically discussed adverse events. As a result, many reviews addressing both safety and efficacy were left out. Our study did not encompass data related to newer treatments like CAR-T cells due to the absence of reviews centered solely on their adverse events. Similarly, bispecific antibodies were not covered extensively. One recent study [66], although emphasizing the safety of bispecific antibodies, also detailed their efficacy, thus not fitting our inclusion criteria. This study did, however, highlight the prevalence of hematologic adverse events and infections. Additionally, a pooled analysis by Mazahreh et al. [67] points out infection risks associated with these innovative drugs, which also exhibit unique adverse event profiles like cytokine release syndrome, neurological issues, and critical infections that require swift intervention [68, 69]. Finally, we did not use GRADE approach to evaluate the quality of all evidence as numerous reviews did not conduct such assessments, and we did not evaluate every primary study ourselves.

### Authors’ conclusions

This overview of systematic reviews provides a thorough description of adverse events of drugs used for the treatment of multiple myeloma. As research in anti-myeloma treatment provides new therapeutic options for patients suffering from the disease, reporting of adverse events should follow closely with efficacy outcomes. Different profiles of toxicity can guide physicians to choose between treatments; however, head-to-head comparison of different treatments is lacking, providing only indirect evidence. The quality of systematic reviews, which are numerous, is not as high as expected. Similarly, reporting was mediocre, failing to adhere to existing appraisal and reporting guidelines, in particular for reporting of adverse events. Current guidelines of reporting for systematic reviews and meta-analysis could be followed for better understanding and appraisal of existing evidence.

### Supplementary information

ESM 1 (PDF 338 kb)

## Data Availability

The data that support the findings of this study are available on request from the authors.
