# Supplementary material for: Reporting of adverse events of treatment interventions in multiple myeloma: an overview of systematic reviews
Source: Ann Hematol. 2023 Nov 8;103(8):2681–97. doi: 10.1007/s00277-023-05517-7 (PMC11283434; doi:10.1007/s00277-023-05517-7)
Supplement: Supplementary file 1 — (PDF 338 kb) [file 277_2023_5517_MOESM1_ESM.pdf]

Reporting of Adverse Events of Treatment Interventions in Multiple Myeloma: An Overview of Systematic Reviews

Annals of Hematology

Maria Mainou, Konstantinos I. Bougioukas, Konstantinos Malandris, Aris Liakos, Philippos Klonizakis, Ioannis Avgerinos, Anna-Betinna Haidich, Apostolos Tsapas

Corresponding author: Maria Mainou, Clinical Research and Evidence-Based Medicine Unit, Second Medical Department, Aristotle University of Thessaloniki, Thessaloniki, Greece; mmainou@auth.gr, ORCID 0000-0002-5775-2241

Appendix

Search Strategy

PUBMED 4.11.22 (downloaded in end note file)

((((((("Multiple Myeloma"[Mesh]) OR "Plasmacytoma"[Mesh]) OR (multiple myeloma OR plasmacytoma OR plasmacytom* OR myelom*)))) AND ((Cochrane Database Syst Rev[Ta] OR Search[tiab] OR Systematic Review[Pt] OR Meta-Analysis[Pt] OR Medline[tiab] OR (Systematic[tiab] AND Review[tiab]) OR Meta-analysis[tiab] OR Meta-analyses[tiab]))) NOT (Animals[MESH] NOT human[MESH]))

Results: 1809

COCHRANE 4.11.22

Search Name: overview

Date Run: 04/11/2022 22:17:06

ID	Search	Hits
#1	MeSH descriptor: [Multiple Myeloma] explode all trees	1807
#2	(multiple myeloma):ti,ab,kw	5840
#3	(myelom*):ti,ab,kw	6889

#4 (plasma cell):ti,ab,kw 13813

#5 systematic review 32213

#6 #1 OR #2 OR #3 OR #4 19623

#7 #6 AND #5 178

From 178:

55 cochrane reviews (imported in endnote)

6 cochrane protocols (imported in endnote)

112 trials

1 editorial

4 clinical answers

Epistemonikos

(title:((title:(multiple myeloma) OR abstract:(multiple myeloma)) AND (title:(systematic review) OR abstract:(systematic review))) OR abstract:((title:(multiple myeloma) OR abstract:(multiple myeloma)) AND (title:(systematic review) OR abstract:(systematic review))))

Results: 393

FINAL endote file before deduplication:

Pubmed 1809

Cochrane reviews 61

Epistemonikos 393

ALL: 2263

Following deduplication in distiller SR (4.11.22) 287 references were excluded.

Final for level 1 Title and Abstract screening: **1976**

List of excluded studies with reasons

Author	Title	Year	Reason for exclusion
**Stansfield, L. C., Gonsalves, W. I., Buadi, F. K.**	The use of novel agents in multiple myeloma patients with hepatic impairment	2015	Wrong study design
**Milrod, C. J., Mann, M., Blevins, F., Hughes, D., Patel, P., Li, K. Y., Lerner, A., Sanchorawala, V., Sloan, J. M.**	Underrepresentation of Black participants and adverse events in clinical trials of lenalidomide for myeloma	2022	Wrong study design
**Colson, K.**	Treatment-related symptom management in patients with multiple myeloma: a review	2015	Wrong study design
**Kumar, A., Galeb, S., Djulbegovic, B.**	Treatment of patients with multiple myeloma: an overview of systematic reviews	2011	Wrong outcomes
**Weisel, K., Doyen, C., Dimopoulos, M. A., Yee, A., Kropff, M., Lahuerta, J. J., Martin, A., Travers, K., Abildgaard, N., Lu, J., Van Droogenbroeck, J., Geraldes, C., Petrini, M., Voillat, L., Vilque, J. P., Voog, E., Facon, T.**	The treatment (TX) of patients (pts) with newly diagnosed multiple myeloma (NDMM) ineligible for transplant: A systematic literature review and network meta-analysis	2015	Wrong outcomes
**Al-Ani, F., Bermejo, J. M., Mateos, M. V., Louzada, M.**	Thromboprophylaxis in multiple myeloma patients treated with lenalidomide - A systematic review	2016	Wrong intervention
**van Nieuwenhuijzen, N., Frunt, R., May, A. M., Minnema, M. C.**	Therapeutic outcome of early-phase clinical trials in multiple myeloma: a meta-analysis	2021	Wrong outcomes
**Lyu, W. W., Zhao, Q. C., Song, D. H., Zhang, J. J., Ding, Z. X., Li, B. Y., Wei, C. M.**	Thalidomide-based Regimens for Elderly and/or Transplant Ineligible Patients with Multiple Myeloma: A Meta-analysis	2016	Wrong outcomes
**Kumar, A., Hozo, I., Wheatley, K., Djulbegovic, B.**	Thalidomide versus bortezomib based regimens as first-line therapy for patients with multiple myeloma: a systematic review	2011	Wrong outcomes
**Gao, M., Kong, Y., Wang, H., Xie, B., Yang, G., Gao, L., Zhang, Y., Zhan, F., Dai, B., Tao, Y., Shi, J.**	Thalidomide treatment for patients with previously untreated multiple myeloma: a meta-analysis of randomized controlled trials	2016	Wrong outcomes
**Alexander, M., Kirsa, S., Mellor, J. D.**	Thalidomide thromboprophylaxis in multiple myeloma: a review of current evidence	2012	Wrong study design
**Kagoya, Y., Nannya, Y., Kurokawa, M.**	Thalidomide maintenance therapy for patients with multiple myeloma: meta-analysis	2012	Wrong outcomes
**El Accaoui, R. N., Shamseddeen, W. A., Taher, A. T.**	Thalidomide and thrombosis. A meta-analysis	2007	Wrong patient population

**Kumar, A., Kharfan-Dabaja, M. A., Glasmacher, A., Djulbegovic, B.**	Tandem versus single autologous hematopoietic cell transplantation for the treatment of multiple myeloma: a systematic review and meta-analysis	2011	Wrong outcomes
**Kumar, A., Kharfan-Dabaja, M. A., Glasmacher, A., Djulbegovic, B.**	Tandem versus single autologous hematopoietic cell transplantation for the treatment of multiple myeloma: a systematic review and meta-analysis	2009	Wrong outcomes
**Armeson, K. E., Hill, E. G., Costa, L. J.**	Tandem autologous vs autologous plus reduced intensity allogeneic transplantation in the upfront management of multiple myeloma: meta-analysis of trials with biological assignment	2013	Wrong outcomes
**Piro, E., Molica, S.**	A systematic review on the use of bortezomib in multiple myeloma patients with renal impairment: what is the published evidence?	2011	Wrong outcomes
**von Lilienfeld-Toal, M., Hahn-Ast, C., Furkert, K., Hoffmann, F., Naumann, R., Bargou, R., Cook, G., Glasmacher, A.**	A systematic review of phase II trials of thalidomide/dexamethasone combination therapy in patients with relapsed or refractory multiple myeloma	2008	Wrong outcomes
**Armoiry, X., Tsertsvadze, A., Connock, M., Melendez-Torres, G. J., Clarke, A.**	Systematic Review and Network Meta-Analysis of Treatment Outcomes for Multiple Myeloma	2017	Wrong outcomes
**Zhang, T., Wang, S., Lin, T., Xie, J., Zhao, L., Liang, Z., Li, Y., Jiang, J.**	Systematic review and meta-analysis of the efficacy and safety of novel monoclonal antibodies for treatment of relapsed/refractory multiple myeloma	2017	Wrong outcomes
**Mu, S. D., Ai, L. S., Qin, Y., Hu, Y.**	Subcutaneous versus Intravenous Bortezomib Administration for Multiple Myeloma Patients: a Meta-analysis	2018	Wrong outcomes
**Ye, Z., Chen, J., Xuan, Z., Yang, W., Chen, J.**	Subcutaneous bortezomib might be standard of care for patients with multiple myeloma: a systematic review and meta-analysis	2019	Wrong outcomes
**Palumbo, A., Waage, A., Hulin, C., Beksac, M., Zweegman, S., Gay, F., Gimsing, P., Leleu, X., Wijermans, P., Sucak, G., Pezzatti, S., Juliusson, G., Pégourié, B., Schaafsma, M., Galli, M., Turesson, I., Kolb, B., van der Holt, B., Baldi, I., Rolke, J., Ciccone, G., Wetterwald, M., Lokhorst, H., Boccadoro, M., Rodon, P., Sonneveld, P.**	Safety of thalidomide in newly diagnosed elderly myeloma patients: a meta-analysis of data from individual patients in six randomized trials	2013	Wrong study design
**Jones, J. R., Pawlyn, C., Davies, F. E., Morgan, G. J.**	The safety of pomalidomide for the treatment of multiple myeloma	2016	Wrong study design
**Jones, J. R., Pawlyn, C., Jackson, G.**	Safety of lenalidomide for maintenance treatment	2021	Wrong study design

**H.**	of patients with multiple myeloma following autologous stem cell transplantation		
**Cengiz Seval, G., Beksac, M.**	The safety of bortezomib for the treatment of multiple myeloma	2018	Wrong study design
**Brioli, A., Mügge, L. O., Hochhaus, A., Von Lilienfeld-Toal, M.**	Safety issues and management of toxicities associated with new treatments for multiple myeloma	2017	Wrong study design
**Tao, Y., Zhou, H., Niu, T.**	Safety and Efficacy Analysis of Selinexor-Based Treatment in Multiple Myeloma, a Meta-Analysis Based on Prospective Clinical Trials	2021	Wrong outcomes
**Roex, G., Timmers, M., Wouters, K., Campillo-Davo, D., Flumens, D., Schroyens, W., Chu, Y., Berneman, Z. N., Lion, E., Luo, F., Anguille, S.**	Safety and clinical efficacy of BCMA CAR-T-cell therapy in multiple myeloma	2020	Wrong outcomes
**Khorochkov, A., Prieto, J., Singh, K. B., Nnadozie, M. C., Shrestha, N., Dominic, J. L., Abdal, M., Abe, R. A. M., Masroor, A., Mohammed, L.**	The Role of Allogeneic Stem Cell Transplantation in Multiple Myeloma: A Systematic Review of the Literature	2021	Wrong outcomes
**Garden, B. C., Nardone, B., Wu, S., West, D. P., Emmanuel, R., Lacouture, M. E.**	Risk of rash associated with lenalidomide in multiple myeloma and myelodisplastic syndrome: A systematic review of the literature and meta-analysis	2012	Wrong patient population
**Laubach, J. P., Moslehi, J. J., Francis, S. A., San Miguel, J. F., Sonneveld, P., Orlowski, R. Z., Moreau, P., Rosiñol, L., Faber, E. A., Jr., Voorhees, P., Mateos, M. V., Marquez, L., Feng, H., Desai, A., van de Velde, H., Elliott, J., Shi, H., Dow, E., Jobanputra, N., Esseltine, D. L., Niculescu, L., Anderson, K. C., Lonial, S., Richardson, P. G.**	A retrospective analysis of 3954 patients in phase 2/3 trials of bortezomib for the treatment of multiple myeloma: towards providing a benchmark for the cardiac safety profile of proteasome inhibition in multiple myeloma	2017	Wrong study design
**Faiman, B., Doss, D., Colson, K., Mangan, P., King, T., Tariman, J. D.**	Renal, GI, and Peripheral Nerves: Evidence-Based Recommendations for the Management of Symptoms and Care for Patients With Multiple Myeloma	2017	Wrong study design
**Vandross, A.**	Proteasome inhibitor-based therapy for treatment of newly diagnosed multiple myeloma	2017	Wrong outcomes
**Neupane, K., Wahab, A., Masood, A., Faraz, T., Bahram, S., Ehsan, H., Hannan, A., Anwer, F.**	Profile and Management of Toxicity of Selinexor and Belantamab Mafodotin for the Treatment of Triple Class Refractory Multiple Myeloma	2021	Wrong study design
**Zoppellaro, G., Veronese, N.,**	Primary thromboembolic prevention in multiple	2018	Wrong intervention

**Granziera, S., Gobbi, L., Stubbs, B., Cohen, A. T.**	myeloma patients: An exploratory meta-analysis on aspirin use		
**Ramsenthaler, C., Kane, P., Gao, W., Siegert, R. J., Edmonds, P. M., Schey, S. A., Higginson, I. J.**	Prevalence of symptoms in patients with multiple myeloma: A systematic review and meta-analysis	2016	Wrong outcomes
**Jessee, J. K.**	Pepaxto: A New Peptide-Drug Conjugate for Heavily Pretreated Relapsed and Refractory Multiple Myeloma	2022	Wrong study design
**Ya, T. A. N., Shuang-nian, X. U., Xi, L. I., Jie-ping, Chen**	Non-myeloablative Stem Cell Transplantation in the Treatment of Multiple Myeloma after First Autologous Stem Cell Transplantation: A Systematic Review	2014	Wrong outcomes
**Botta, C., Ciliberto, D., Rossi, M., Staropoli, N., Cucè, M., Galeano, T., Tagliaferri, P., Tassone, P.**	Network meta-analysis of randomized trials in multiple myeloma: efficacy and safety in relapsed/refractory patients	2017	Wrong outcomes
**Botta, C., Gigliotta, E., Paiva, B., Anselmo, R., Santoro, M., Otero, P. R., Carlisi, M., Conticello, C., Romano, A., Solimando, A. G., Cerchione, C., Vià, M. D., Bolli, N., Correale, P., Di Raimondo, F., Gentile, M., San Miguel, J., Siragusa, S.**	Network meta-analysis of randomized trials in multiple myeloma: Efficacy and safety in frontline therapy for patients not eligible for transplant	2022	Wrong outcomes
**Piechotta, V., Jakob, T., Langer, P., Monsef, I., Scheid, C., Estcourt, L. J., Ocheni, S., Theurich, S., Kuhr, K., Scheckel, B., et al.**	Multiple drug combinations of bortezomib, lenalidomide, and thalidomide for first-line treatment in adults with transplant-ineligible multiple myeloma: a network meta-analysis	2019	Wrong outcomes
**Zheng, Y., Shen, H., Xu, L., Feng, J., Tang, H., Zhang, N., Chen, X., Gao, G.**	Monoclonal Antibodies versus Histone Deacetylase Inhibitors in Combination with Bortezomib or Lenalidomide plus Dexamethasone for the Treatment of Relapsed or Refractory Multiple Myeloma: An Indirect-Comparison Meta-Analysis of Randomized Controlled Trials	2018	Wrong outcomes
**Knopf, K. B., Duh, M. S., Lafeuille, M. H., Gravel, J., Lefebvre, P., Niculescu, L., Ba-Mancini, A., Ma, E., Shi, H., Comenzo, R. L.**	Meta-analysis of the efficacy and safety of bortezomib re-treatment in patients with multiple myeloma	2014	Wrong outcomes
**Goel, L., Gupta, P., Pahuja, M.**	Mechanistic Involvement of Inflammation in Bortezomib-induced Peripheral Neuropathy	2022	Wrong study design
**Wang Huan, H. Y., Wu, T. X.**	Lenalidomide plus dexamethasone for relapsed or refractory multiple myeloma: a systematic review	2011	Wrong outcomes
**Liu, Z. Q., Xia, H. L., Li, C. J., Xia, L.**	[Incidence and Risk of Peripheral Neuropathy	2019	Non-english language

	Caused by Intravenous and Subcutaneous Injection of Bortezomib]		
**Oostvogels, R., Venema, S. M. U., de Witte, M., Raymakers, R., Kuball, J., Kröger, N., Minnema, M. C.**	In search of the optimal platform for Post-Allogeneic SCT immunotherapy in relapsed multiple myeloma: a systematic review	2017	Wrong study design
**Kumar, S. K., Laubach, J. P., Giove, T. J., Quick, M., Neuwirth, R., Yung, G., Rajkumar, S. V., Richardson, P. G.**	Impact of concomitant dexamethasone dosing schedule on bortezomib-induced peripheral neuropathy in multiple myeloma	2017	Wrong study design
**Mohyuddin, G. R., Sigle, M., Chandrasekar, V. T., Aziz, M., Abdallah, A. O., Shune, L., McClune, B.**	Impact of anti-CD38 therapy in multiple myeloma with high-risk cytogenetics: systematic review and meta-analysis	2020	Wrong outcomes
**Koreth, J., Cutler, C. S., Djulbegovic, B., Behl, R., Schlossman, R. L., Munshi, N. C., Richardson, P. G., Anderson, K. C., Soiffer, R. J., Alyea, E. P.**	High-dose therapy with single autologous transplantation versus chemotherapy for newly diagnosed multiple myeloma: A systematic review and meta-analysis of randomized controlled trials	2007	Wrong outcomes
**Zheng, H., Yang, F.**	Gemcitabine in treating patients with refractory or relapsed multiple myeloma	2014	Wrong outcomes
**Sekine, L., Ziegelmann, P. K., Manica, D., da Fonte Pithan, C., Sosnoski, M., Morais, V. D., Falcetta, F. S., Ribeiro, M. R., Salazar, A. P., Ribeiro, R. A.**	Frontline treatment for transplant-eligible Multiple Myeloma: a 6,474 patients network meta-analysis	2019	Wrong outcomes
**Frauke, Naumann-Winter, Alexander, Greb, Peter, Borchmann, Julia, Bohlius, Andreas, Engert, Roland, Schnell**	First-line tandem high-dose chemotherapy and autologous stem cell transplantation versus single high-dose chemotherapy and autologous stem cell transplantation in multiple myeloma, a systematic review of controlled studies	2012	Wrong outcomes
**Rodríguez, Myriam Riam Lucia, Buendía, Jefferson, Combariza, Juan, Casas, Claudia Patricia, Carvajal, Arturo Marti, Ramírez, Henry Becerra, Cardona, Andrés Felipe, Reveiz, Ludovick**	First line therapy for patients with newly diagnosed multiple myeloma ineligible for autologous stem cell transplantation: a systematic review and meta-analysis (hemo-oncolgroup study)	2012	Wrong outcomes
**Parrondo, R. D., Reljic, T., Iqbal, M., Ayala, E., Kharfan-Dabaja, M. A., Kumar, A., Murthy, H. S.**	Efficacy of Proteasome Inhibitor-Based Maintenance following Autologous Transplantation in Multiple Myeloma: a systematic review and meta-analysis	2021	Wrong outcomes
**Menon, T., Kataria, S., Adhikari, R., Khan, H., Khalid, M. Z.,**	Efficacy of Daratumumab-Based Regimens Compared to Standard of Care in Transplant-Eligible	2021	Wrong outcomes

**Saeeduddin, M. O., Taj, S., Rehman, U., Tekin, A., Singh, R.**	Multiple Myeloma: A Meta-Analysis		
**Atkins, J., Fowler, S. A., Tuuli, M., James, A. S., Wildes, T. M.**	Efficacy of bortezomib-based regimens with or without an immunomodulatory agent in older adults with multiple myeloma: A systematic review and meta-analysis	2013	Wrong outcomes
**Imtiaz, H., Khan, M., Ehsan, H., Wahab, A., Rafae, A., Khan, A. Y., Jamil, A., Sana, M. K., Jamal, A., Ali, T. J., Ansar, I., Khan, M. M., Khouri, J., Anwer, F.**	Efficacy and Toxicity Profile of Carfilzomib-Based Regimens for Treatment of Newly Diagnosed Multiple Myeloma: A Systematic Review	2021	Wrong outcomes
**Mushtaq, A., Kapoor, V., Latif, A., Iftikhar, A., Zahid, U., McBride, A., Abraham, I., Riaz, I. B., Anwer, F.**	Efficacy and toxicity profile of carfilzomib based regimens for treatment of multiple myeloma: A systematic review	2018	Wrong outcomes
**Gao, X., Shen, L., Li, X., Liu, J.**	Efficacy and toxicity of histone deacetylase inhibitors in relapsed/refractory multiple myeloma: Systematic review and meta-analysis of clinical trials	2019	Wrong outcomes
**Xie, C., Wei, M., Yang, F., Liu, Q., Wu, F., Huang, J.**	Efficacy and toxicity of carfilzomib- or bortezomib-based regimens for treatment of transplant-ineligible patients with newly diagnosed multiple myeloma: A meta-analysis	2022	Wrong outcomes
**Hu, B., Zhou, Q., Wu, T., Zhuang, L., Yi, L., Cao, J., Yang, X., Wang, J.**	Efficacy and safety of subcutaneous versus intravenous bortezomib in multiple myeloma: a meta-analysis	2017	Wrong outcomes
**Zhang, S., Li, J., Liu, P.**	Efficacy and safety of subcutaneous bortezomib versus intravenous bortezomib in patients with multiple myeloma: A systematic review and meta-analysis	2018	Wrong outcomes
**Sopeña, M., Clavero, E. M., Villa, P., Martínez-López, J.**	Efficacy and safety of reduced-intensity induction therapy with a bortezomib-based regimen in elderly patients with multiple myeloma	2012	Wrong study design
**Lin, J., Chen, J., Zeng, Z., Qiu, D., Wang, J.**	Efficacy and safety of pegylated liposomal doxorubicin for multiple myeloma: A systematic review and meta-analysis of randomized controlled trials	2017	Wrong outcomes
**Liu, J. D., Sun, C. Y., Tang, L., Wu, Y. Y., Wang, Q. Y., Hu, B., Hu, Y.**	Efficacy and Safety of Panobinostat in Relapsed or/and Refractory Multiple Myeloma: Meta Analyses of Clinical Trials and Systematic Review	2016	Wrong outcomes
**Qiao, S. K., Guo, X. N., Ren, J. H., Ren, H. Y.**	Efficacy and Safety of Lenalidomide in the Treatment of Multiple Myeloma: A Systematic Review and Meta-analysis of Randomized Controlled Trials	2015	Wrong outcomes

**Zhong, J., Zhang, X., Liu, M.**	The efficacy and safety of lenalidomide in the treatment of multiple myeloma patients after allo-hematopoietic stem-cell transplantation: a systematic review and meta-analysis	2021	Wrong outcomes
**Chen, H., Wang, Y., Shao, C., Sun, C., Zheng, C.**	Efficacy and safety of ixazomib maintenance therapy for patients with multiple myeloma: a meta-analysis	2021	Wrong outcomes
**Wang, Y., Li, Y., Chai, Y.**	Efficacy and safety of daratumumab in the treatment of multiple myeloma: a systematic review and meta-analysis	2021	Wrong outcomes
**Li, J., Tang, Y., Huang, Z.**	Efficacy and safety of chimeric antigen receptor (CAR)-T cell therapy in the treatment of relapsed and refractory multiple myeloma: a systematic-review and meta-analysis of clinical trials	2022	Wrong outcomes
**Yang, Q., Li, X., Zhang, F., Yang, Q., Zhou, W., Liu, J.**	Efficacy and Safety of CAR-T Therapy for Relapse or Refractory Multiple Myeloma: A systematic review and meta-analysis	2021	Wrong outcomes
**Xiang, X., He, Q., Ou, Y., Wang, W., Wu, Y.**	Efficacy and Safety of CAR-Modified T Cell Therapy in Patients with Relapsed or Refractory Multiple Myeloma: A Meta-Analysis of Prospective Clinical Trials	2020	Wrong outcomes
**Luo, T., Xia, H. L.**	[Efficacy and Safety of Carfilzomib in the Treatment of Multiple Myeloma ： A Systematic Evaluation]	2019	Wrong outcomes
**Shah, C., Bishnoi, R., Wang, Y., Zou, F., Bejjanki, H., Master, S., Moreb, J. S.**	Efficacy and safety of carfilzomib in relapsed and/or refractory multiple myeloma: Systematic review and meta-analysis of 14 trials	2018	Wrong outcomes
**Georgoulis, V., Haidich, A. B., Bougioukas, K. I., Hatzimichael, E.**	Efficacy and safety of carfilzomib for the treatment of multiple myeloma: An overview of systematic reviews	2022	Wrong outcomes
**Aguiar, P. M., de Mendonça Lima, T., Colleoni, G. W. B., Storpirtis, S.**	Efficacy and safety of bortezomib, thalidomide, and lenalidomide in multiple myeloma: An overview of systematic reviews with meta-analyses	2017	Wrong outcomes
**Sun, C. Y., Li, J. Y., Chu, Z. B., Zhang, L., Chen, L., Hu, Y.**	Efficacy and safety of bortezomib maintenance in patients with newly diagnosed multiple myeloma: a meta-analysis	2017	Wrong outcomes
**Mateos, M. V., San-Miguel, J., Goldschmidt, H., Sonneveld, P., Dimopoulos, M. A., Heeg, B., Hashim, M., Deraedt, W., Hu, P., Lam, A., He, J.**	The effects of different schedules of bortezomib, melphalan, and prednisone for patients with newly diagnosed multiple myeloma who are transplant ineligible: a matching-adjusted indirect comparison	2020	Wrong outcomes
**Łopuch, S., Kawalec, P.,**	Effectiveness of targeted therapy as monotherapy or combined therapy in patients with relapsed or	2015	Wrong outcomes

**Wiśniewska, N.**	refractory multiple myeloma: a systematic review and meta-analysis		
**He, Y., Wheatley, K., Clark, O., Glasmacher, A., Ross, H., Djulbegovic, B.**	Early versus deferred treatment for early stage multiple myeloma	2003	Wrong outcomes
**Zhao, A. L., Shen, K. N., Wang, J. N., Huo, L. Q., Li, J., Cao, X. X.**	Early or deferred treatment of smoldering multiple myeloma: a meta-analysis on randomized controlled studies	2019	Wrong outcomes
**Xu, W., Li, D., Sun, Y., Ran, X., Wang, B., Wu, W., Sheng, Z., Liu, L.**	Daratumumab added to standard of care in patients with newly diagnosed multiple myeloma: A network meta-analysis	2019	Wrong outcomes
**Wang, L., Xiang, H., Yan, Y., Deng, Z., Li, H., Li, X., Liu, J.**	Comparison of the efficiency, safety, and survival outcomes in two stem cell mobilization regimens with cyclophosphamide plus G-CSF or G-CSF alone in multiple myeloma: a meta-analysis	2021	Wrong outcomes
**Ye, W., Wu, X., Liu, X., Zheng, X., Deng, J., Gong, Y.**	Comparison of monoclonal antibodies targeting CD38, SLAMF7 and PD-1/PD-L1 in combination with Bortezomib/Immunomodulators plus dexamethasone/prednisone for the treatment of multiple myeloma: an indirect-comparison Meta-analysis of randomised controlled trials	2021	Wrong outcomes
**Cengiz Seval, G., Beksac, M.**	A comparative safety review of histone deacetylase inhibitors for the treatment of myeloma	2019	Wrong study design
	Combination chemotherapy versus melphalan plus prednisone as treatment for multiple myeloma: an overview of 6,633 patients from 27 randomized trials. Myeloma Trialists' Collaborative Group	1998	Wrong outcomes
**Mohyuddin, G. R., Rooney, A., Balmaceda, N., Aziz, M., Sborov, D. W., McClune, B., Kumar, S. K.**	Chimeric antigen receptor T-cell therapy in multiple myeloma: a systematic review and meta-analysis of 950 patients	2021	Wrong outcomes
**Sheng, Z., Li, G., Li, B., Liu, Y., Wang, L.**	Carfilzomib-containing combinations as frontline therapy for multiple myeloma: A meta-analysis of 13 trials	2017	Wrong outcomes
**Camilli, M., La Vecchia, G., Lillo, R., Iannaccone, G., Lamendola, P., Montone, R. A., Hohaus, S., Aspromonte, N., Massetti, M., Lanza, G. A., Crea, F., Graziani, F., Lombardo, A.**	Cardiovascular involvement in patients affected by multiple myeloma: a comprehensive review of recent advances	2021	Wrong study design
**Huang, H., Zhou, L., Peng, L., Fu, W., Zhang, C., Hou, J.**	Bortezomib-thalidomide-based regimens improved clinical outcomes without increasing toxicity as induction treatment for untreated multiple	2014	Wrong outcomes

	myeloma: a meta-analysis of phase III randomized controlled trials		
**Leiba, M., Kedmi, M., Duek, A., Freidman, T., Weiss, M., Leiba, R., Nagler, A., Avigdor, A.**	Bortezomib-cyclophosphamide-dexamethasone (VCD) versus bortezomib-thalidomide-dexamethasone (VTD) -based regimens as induction therapies in newly diagnosed transplant eligible patients with multiple myeloma: a meta-analysis	2014	Wrong outcomes
**Nooka, A. K., Kaufman, J. L., Behera, M., Langston, A., Waller, E. K., Flowers, C. R., Gleason, C., Boise, L. H., Lonial, S.**	Bortezomib-containing induction regimens in transplant-eligible myeloma patients: a meta-analysis of phase 3 randomized clinical trials	2013	Wrong outcomes
**Wang, L., Xu, Y. L., Zhang, X. Q.**	Bortezomib in combination with thalidomide or lenalidomide or doxorubicin regimens for the treatment of multiple myeloma: a meta-analysis of 14 randomized controlled trials	2014	Wrong outcomes
**Scott, K., Hayden, P. J., Will, A., Wheatley, K., Coyne, I.**	Bortezomib for the treatment of multiple myeloma	2016	Wrong outcomes
**Zeng, Z., Lin, J., Chen, J.**	Bortezomib for patients with previously untreated multiple myeloma: a systematic review and meta-analysis of randomized controlled trials (Provisional abstract)	2013	Wrong outcomes
**Zou, Y., Lin, M., Sheng, Z., Niu, S.**	Bortezomib and lenalidomide as front-line therapy for multiple myeloma	2014	Wrong outcomes
**Mhaskar, R., Kumar, A., Miladinovic, B., Djulbegovic, B.**	Bisphosphonates in multiple myeloma: an updated network meta-analysis	2017	Wrong outcomes
**Mhaskar, R., Redzepovic, J., Wheatley, K., Clark, O. A., Miladinovic, B., Glasmacher, A., Kumar, A., Djulbegovic, B.**	Bisphosphonates in multiple myeloma: a network meta-analysis	2012	Wrong outcomes
**Mhaskar, R., Redzepovic, J., Wheatley, K., Clark, O. A., Miladinovic, B., Glasmacher, A., Kumar, A., Djulbegovic, B.**	Bisphosphonates in multiple myeloma	2010	Wrong outcomes
**Djulbegovic, B., Wheatley, K., Ross, J., Clark, O., Bos, G., Goldschmidt, H., Cremer, F., Alsina, M., Glasmacher, A.**	Bisphosphonates in multiple myeloma	2002	Wrong outcomes
**Sarasquete, M. E., González, M., San Miguel, J. F., García-Sanz, R.**	Bisphosphonate-related osteonecrosis: genetic and acquired risk factors	2009	Wrong study design
**He, X., Yang, K., Chen, P., Liu, B., Zhang, Y., Wang, F., Guo, Z., Liu, X.,**	Arsenic trioxide-based therapy in relapsed/refractory multiple myeloma patients: a	2014	Wrong outcomes

**Lou, J., Chen, H.**	meta-analysis and systematic review		
**Kahale, L. A., Matar, C. F., Tsolakian, I., Hakoum, M. B., Yosuico, V. E., Terrenato, I., Sperati, F., Barba, M., Hicks, L. K., Schünemann, H., Akl, E. A.**	Antithrombotic therapy for ambulatory patients with multiple myeloma receiving immunomodulatory agents	2021	Wrong outcomes
**Mohyuddin, G. R., Aziz, M., McClune, B., Abdallah, A. O., Qazilbash, M.**	Antibiotic Prophylaxis for Patients with Newly Diagnosed Multiple Myeloma: Systematic Review and Meta-Analysis	2020	Wrong intervention
**Yin, X., Tang, L., Fan, F., Jiang, Q., Sun, C., Hu, Y.**	Allogeneic stem-cell transplantation for multiple myeloma: a systematic review and meta-analysis from 2007 to 2017	2018	Wrong outcomes
**Wang, Y., Lv, B., Li, K., Zhang, A., Liu, H.**	Adjuvant immunotherapy of dendritic cells and cytokine-induced killer cells is safe and enhances chemotherapy efficacy for multiple myeloma in China: a meta-analysis of clinical trials	2017	Wrong outcomes
**Kiss, S., Gede, N., Hegyi, P., Nagy, B., Deák, R., Dembrovszky, F., Bunduc, S., Erőss, B., Leiner, T., Szakács, Z., Alizadeh, H.**	Addition of daratumumab to multiple myeloma backbone regimens significantly improves clinical outcomes: a systematic review and meta-analysis of randomised controlled trials	2021	Wrong outcomes
